# Non-Ionic Surfactant Recovery in Surfactant Enhancement Aquifer Remediation Effluent with Chlorobenzenes by Semivolatile Chlorinated Organic Compounds Volatilization

**DOI:** 10.3390/ijerph19127547

**Published:** 2022-06-20

**Authors:** Patricia Sáez, Aurora Santos, Raúl García-Cervilla, Arturo Romero, David Lorenzo

**Affiliations:** Chemical Engineering and Materials Department, Complutense University of Madrid, 28040 Madrid, Spain; patrisae@ucm.es (P.S.); aursan@quim.ucm.es (A.S.); raugar05@ucm.es (R.G.-C.); aromeros@quim.ucm.es (A.R.)

**Keywords:** surfactant, chlorinated organic compounds, volatilization, emulsion treatment, SEAR emulsion treatment

## Abstract

Surfactant enhanced aquifer remediation is a common treatment to remediate polluted sites with the inconvenience that the effluent generated must be treated. In this work, a complex mixture of chlorobenzene and dichlorobenzenes in a non-ionic surfactant emulsion has been carried out by volatilization. Since this techhnique is strongly affected by the presence of the surfactant, modifying the vapour pressure, $P_{v}^{0},$ and activity coefficient, γ, a correlation between $P_{v_{j}}^{0}\gamma_{j}$ and surfactant concentration and temperature was proposed for each compound, employing the Surface Response Methodology (RSM). Volatilization experiments were carried out at different temperatures and gas flow rates. A good agreement between experimental and predicted remaining SVCOCs during the air stripping process was obtained, validating the thermodynamic parameters obtained with RSM. Regarding the results of volatilization, at 60 °C 80% of SVCOCs were removed after 6 h, and the surfactant capacity was almost completely recovered so the solution can be recycled in soil flushing.

## 1. Introduction

The contamination of soil and groundwater by, among others, chlorinated organic compounds has become a severe environmental issue [1]. These pollutants present a high oil/water distribution coefficient and a low water solubility. The accidental release or intentional dumping of hydrophobic organic liquid phases into the environment has resulted in a separate liquid phase, termed non-aqueous phase liquids (or NAPLs), that persists in the subsurface [2].

One of the remediation treatments that was successfully applied to remove the NAPLs mass in the subsurface in a short time is Surfactant Enhancement Aquifer Remediation (SEAR) [3,4]. The surfactants enhance the removal of pollutants through two mechanisms: solubilization and mobilization. The amphoteric properties of the surfactants that reduce interface tension facilitate the transport of hydrophobic pollutants to the aqueous phase [5]. This technique involves injecting an aqueous solution containing a surfactant into the contaminated area with further extraction of the fluid injected containing the solubilized–mobilized pollutants [4,6,7].

The SEAR technique presents significant benefits compared to other technologies, such as pump and treat [8], since it increases the efficiency in remediating areas contaminated with NAPLs. However, the SEAR process moves the contamination from the subsurface into the aqueous phase but does not eliminate the contaminant, resulting in a secondary contamination [3]. The emulsion extracted is composed of a complex mixture of organic compounds and the surfactant used and must be treated to eliminate the organic pollutants. Moreover, the recovery of the surfactant is highly desired in a circular economy perspective. 

Selective oxidation of organic contaminants in the emulsion has been proposed and tested successfully [9,10,11,12]. Still, the cost of reagents and the loss of surfactant capacity after treatment can decrease the sustainability of this treatment. Organic compounds are not mineralized, and the loss of surfactant stability is associated with the unproductive consumption of the oxidant by the surfactant [13]. The more refractory the contaminant is to oxidation, the higher the unproductive consumption of the oxidant. Selective adsorption of the organic pollutants in the emulsion on activated carbon and selective organic compound retention by membranes have also been proposed to treat contaminated emulsions [10,14,15,16,17]. However, membrane fouling and surfactant adsorption decrease the effectiveness of these methods. 

The air stripping of volatile or semivolatile chlorinated organic compounds in soil (VCOCs–SVCOCs) has been reported at the field scale [18,19,20]. Still, this topic has been scarcely studied in the scientific literature. This technique transfers the volatile compound from an aqueous solution to an air stream. The volatilized chlorinated organic compound (COC) can be more selectively adsorbed on activated carbon. This process is effective when the organic compounds are volatile or semivolatile [21]. However, the volatility of COCs in the emulsion should be affected by the surfactant presence, being that this topic is not studied in the literature. Moreover, the loss of surfactant capacity during air stripping has not been considered.

This work aims to study and model an air stripping process to eliminate a mixture of chlorobenzene and dichlorobenzene isomers in a non-ionic surfactant emulsion (Emulse-3^®^ as surfactant). These chlorinated compounds are often used as solvents or reagents, and leaks from storage tanks result in soil and groundwater contamination [22,23,24].

The temperature applied during the volatilization process could modify the surfactant stability, and this aspect has also been studied in this work. The volatility of chlorinated organic compounds in the emulsion has also been determined. From our knowledge this topic has not been studied in the literature. The influence of the surfactant presence in the aqueous phase on SVCOCs volatility has been analyzed determining the $P_{v_{j}}^{0}\gamma_{j}$ values at different surfactant and SVCOCs concentration and temperatures. The volatilization of SVCOCs in the emulsion by air streaming at different conditions has been modelled and validated. 

## 2. Materials and Methods

### 2.1. Chemicals

The mixture of SVCOCs used in this work consisted of chlorobenzene (CB), 1,2-dichlorobenzene (1,2-DCB), and 1,4-dichlorobenzene (1,4-DCB) prepared from commercial compounds (Sigma Aldrich, Darmstadt, Germany, analytical grade). The distribution of each compound, expressed as molar percentage, was 53% of CB, 29% of 1,2-DCB, and 18% of 1,4-DCB. 

The quantification of SVCOCs was carried out using calibration curves prepared from standard samples of known concentration in methanol from commercial compounds (Sigma Aldrich, analytical grade). Additionally, the limonene ((R)-(+)-Limonene, Sigma Aldrich) (cosolvent of surfactant) was also calibrated. Bicyclohexyl ($C_{12}H_{22}$, Sigma Aldrich) and tetrachloroethane ($C_{2}H_{2}Cl_{4}$, Sigma Aldrich) were used as internal standards (ISTD) for quantification by gas chromatography (GC). The chromatographic method is explained elsewhere [25].

The surfactant selected to carry out the experiments was E-Mulse 3^®^ (E3) (EthicalChem), which is a non-ionic surfactant with a critical micelle concentration (CMC), measured of $80\;\mathrm{mg}\cdot L^{-1}$. This surfactant has been successfully applied in the solubilization of 28 different chlorinated organic compounds present in a real dense non-aqueous phase liquid (DNAPL) to the aqueous phase, including CB and DCBs [26].

The air employed to perform the air stripping experiments was supplied by Carburos Metálicos, with an air purity of 99.999% ($Alphagaz^{TM}\;1\;AR,\;Air\;Liquid$). The aqueous solutions were prepared with high-purity water from a Millipore Direct-Q system with resistivity >18 MΩ·cm at 25 °C. 

### 2.2. Experimental Procedure

The experimental procedure comprised three experiment blocks. In the first one (B1), the surfactant stability was studied at different temperatures. In the second one (B2), the influence of surfactant concentration, temperature, and chlorinated compounds concentration on each COC (CB, 1,2-DCB, and 1,4-DCB) volatility was analyzed. The product of vapour pressure by the activity coefficient $\left(P_{v_{j}}^{o}\gamma_{j}\right)$ was obtained and correlated with the variables studied. Finally, SVCOCs volatilization in the emulsion (B3) was carried out by passing an airstream through the aqueous emulsion at several temperatures and air flow rates.

#### 2.2.1. Surfactant Stability (B1)

Surfactant stability experiments were performed in batch mode employing sealed 20 mL glass vials for gas chromatography without headspace closed with PTFE (polytetrafluoroethylene) caps in the absence and presence of SVCOCs. In the last case, the aqueous phase was saturated with the mixture of VCOCs, adding the corresponding amount of SVCOCs to obtain a saturated emulsion of organic phase in the aqueous surfactant emulsion. The amount of VCOCs added was calculated from the molar solubilization ratio MSR (amount of SVCOCs that can be solubilized in the surfactant solution when saturation is reached) obtained elsewhere for a complex mixture of chlorinated compounds in this surfactant [26] (MSR =$\;4.33\;\mathrm{mmol}\;\mathrm{SVCOCs}\cdot g_{\mathrm{surf}}^{-1}$). The emulsions were agitated during 4 h and left to settle 24 h without agitation, checking the total solubilization of VCOCs added.

The vials were prepared with 19 mL of surfactant emulsion (with or without contaminant). Vials were heated in a thermostatic bath to obtain the desired temperature (25–60 °C) and agitated using a magnetic stirrer for up to 48 h. Zero time was considered when the desired temperature was reached. The experimental conditions are summarized in Table 1 (runs E1 to E6).

The remaining surfactant concentration was analyzed by sacrificing a vial at the corresponding time, including zero. In the experiments carried using emulsion saturated in SVCOCs, the remaining surfactant concentration was calculated from the remaining SVCOCs in solution, considering the MSR value as shown in Equation (1).

(1) $$C_{s}\left({g\;L}^{-1}\right)=\frac{C_{SVCOCs\;}\left(\mathrm{mM}\right)}{4.33\;\left(\frac{{\mathrm{mmol}}_{\mathrm{SVCOCs}}}{g_{\mathrm{surf}}}\right)}$$

where *C_S_* is the surfactant concentration ($g_{\mathrm{surf}}\cdot L^{-1})$, $C_{SVCOCs}$ is the concentration of the sum of the three chlorinated organic compounds $\left({\mathrm{mmol}}_{\mathrm{VCOCs}}\cdot L^{-1}\right),\;$ and 4.33 is the solubilization mass solubilization ratio of E3 with the chlorinated compounds in ${\mathrm{mmol}}_{\mathrm{VCOCs}}\cdot g_{\mathrm{surf}}^{-1}$.

In the absence of pollutant, the remaining surfactant concentration at each time was calculated by dissolving 1,2,4-trichlorobenzene (1,2,4-TCB), measuring the solubilized concentration of this compound in the aqueous phase and using Equation (1) taking into account that $MSR_{1,2,4-TCB}=4.33\;{\mathrm{mmol}}_{1,2,4-\mathrm{TCB}}\;\cdot g_{\mathrm{surf}}^{-1}$. All the experiments were replicated, with differences among experimental results lower than 7%. The average values were used as the experimental results.

#### 2.2.2. B2. Estimation of $P_{v_{j}}^{o}\gamma_{j}$ (B2)

This set of experiments was proposed to estimate the product of $P_{v_{j}}^{o}\gamma_{j}$, of CB, 1,2-DCB, and 1,4-DCB in the presence of surfactant at several temperatures.

Firstly, certain amounts of CB and DCB isomers were solubilized in an aqueous solution of the surfactant at the corresponding concentration. The emulsion was prepared in 100 mL flasks, without headspace, to avoid the volatile loss. Surfactant E3 concentration ranged from $1.5\;g\cdot L^{-1}\;\mathrm{to}\;15\;g\cdot L^{-1}$. The amount of SVCOCs was varied from $3.1\;\mathrm{mmol}\cdot L^{-1}\;\mathrm{to}\;62.8\;\mathrm{mmol}\cdot L^{-1}$ being always less than that required for saturation at the surfactant concentration used. After 2 h of agitation, the solution was settled, checking that all the SVCOCs added were dissolved by GC-FID. Following, 10 mL of the emulsion was transferred to 20 mL glass vials for gas chromatography, closed, and agitated at different temperatures (30–60 °C) for 1 h in the incubator of HeadSpace Gas Chromatography (HS-GC), Agilent GC Sampler 120. This time was enough to reach the equilibrium between liquid and vapour phases generated. The SVCOCs in the vapour phase were analyzed by HeadSpace coupled with GC/FID/ECD. Table 1 summarizes the conditions of the experiments carried out (runs P1 to P4).

#### 2.2.3. Volatlization Tests (B3)

**Figure 1:**
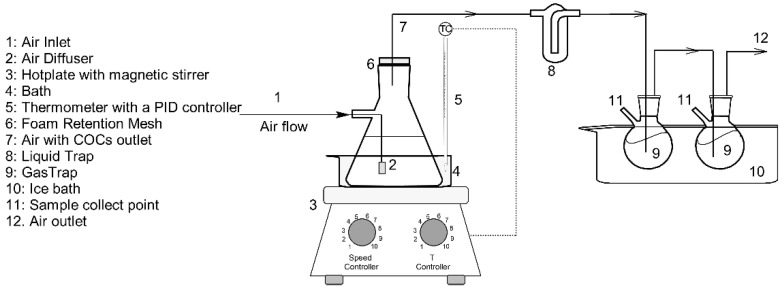
Scheme of the installation used for volatilization tests.

The volatilization of volatile chlorinated organic compounds from aqueous surfactant emulsion passing an air flow rate was performed in the experimental setup schematized in Figure 1. The air was bubbled in the aqueous emulsion from pressurized air in a cylinder, and the gas flowrate was controlled using a mass flow controller ($EL-FLOW^{®}$ Select Series Mass Flow Meters/Controllers for gases, ${\mathrm{Bronkhorst}}^{®}$). The air was introduced into the emulsion by a diffuser to favour the gas–liquid equilibrium. The system temperature was regulated with a hotplate (IKA C-MAG HS 7) and controlled with a thermometer with a PID (Proportional Integral Derivative) controller (IKA ETS-D5). The gas phase leaving the emulsion was saturated in SVCOCs, conducted through an iron mesh (100 µm) to prevent excessive foams formation, and bubbled in MetOH, which acted like a liquid trap. The MetOH traps were introduced into an ice bath to avoid volatile loss. Samples were taken periodically from the emulsion to monitor the remaining amount of VCOCs and the surfactant concentration. The surfactant concentration in the aqueous phase with time was measured employing 1,2,4-TCB as explained in B1. 

The volatilization experiments were maintained for 8 h when the airflow was stopped. The experiments were carried out at $3.5\;g\cdot L^{-1}$ of surfactant, $23.5\;\mathrm{mmol}\cdot L^{-1}$ of initial VCOCs concentration in a volume of 0.30 L. Table 1 provides a summary of the conditions of the experiments carried out (runs V1 to V3). 

### 2.3. SVCOCs Analysis

The concentration of SVCOCs in emulsion was analyzed by gas chromatography (Agilent 8860) with autosampler (Agilent GC Sampler 120) coupled with a flame ionization detector and an electron capture detector (GC-FID/ECD). The column was Agilent HP5-MSUI (19091S-433UI, 30 m × 0.25 mm ID × 0.25 µm). Two microliters of samples were injected using helium as carrier gas (flow rate of $2.9\;\mathrm{mL}\cdot {\;\min}^{-1}$). The GC injection port temperature was set at 250 °C, and GC oven worked at a programmed temperature gradient, starting at 80 °C and raising the temperature at a rate of $15\;{}^{\circ}C\cdot \min^{-1}$ until 180 °C, and then keeping it constant for 15 min. Additionally, a split ratio of 10:1 was employed in the analysis. The samples were previously diluted 1:10 with methanol.

The SVCOCs concentrations in the vapour phase in B2 experiments were measured by HeadSpace Gas Chromatography (HS-GC). Twenty millileter glass vials for gas chromatography closed with PTFE caps, were filled with 10 mL of the emulsion of SVCOCs mixture. The vials were agitated and heated at constant temperature (depending on the experimental conditions tested from 30 °C to 60 °C) for 1 h, ensuring the equilibrium between liquid and vapour was reached. After this time, 2.5 mL of the vapour phase was to the GC using a 10:1 split ratio. The column and the method conditions employed were the same as described for analyzing SVCOCs dissolved. More details of the method are shown in Table S1.

## 3. Results and Discussions

### 3.1. Surfactant Stability

**Figure 2:**
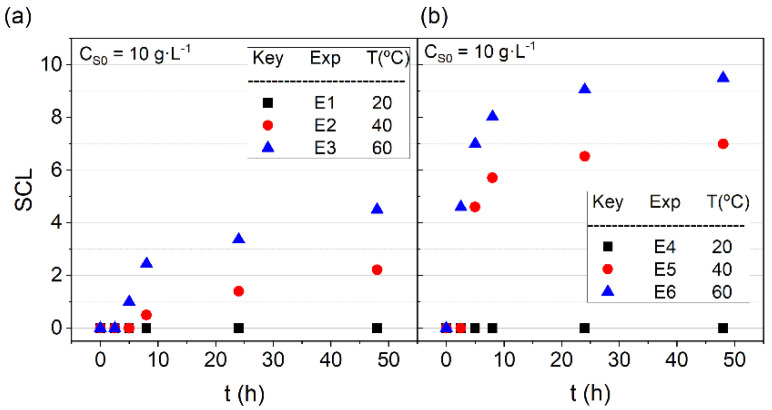
SCL profiles with time. $C_{So}=$ 10 $g\cdot L^{-1}$, temperature = (20, 40 and 60) °C (**a**) absence of SVCOCs; (**b**) presence of SVCOCs.

The experiments summarized in Table 1 for the B1 experiment set were carried out to study the surfactant stability. The results obtained are expressed as the evolutions of Surfactant Capacity Loss (SCL) with the time. SCL is calculated with Equation (2) and refers to the fractional remaining surfactant capacity express in percentage.

(2) $$SCL=\left(1-\frac{C_{S}}{C_{So}}\right)\cdot 100$$

where $C_{S}$ is the surfactant concentration at each time by Equation (1)$\;\left(g\cdot L^{-1}\right)$ and $C_{S0}$ is the initial surfactant concentration $\left(g\cdot L^{-1}\right)$. Figure 2 shows the results obtained from the experiments without SVCOCs (a) and with SVCOCs (b).

As shown in Figure 2, the SCL obtained for all the experiments is lower than 10% after 40 h, even at the maximum temperature used, 60 °C, indicating the stability of the surfactant in the operation range studied. The presence of SVCOCs slightly modifies the surfactant stability. The SCL values without SVCOCs range from 2% to 4% at 40 °C and 60 °C, respectively. In the presence of SVCOCs, the SCL ranges from 7 % to 9% at 40 °C and 60 °C, respectively. The differences found in the experiment carried out with and without SVCOCs can be attributed to the modification of the partial pressure of the surfactant, being higher in the presence of organic compounds. 

Nevertheless, the SCL is always less than 10% in the time interval, and the temperature range studied being considered negligible. Therefore, the active surfactant concentration with time corresponds to the the initial value, $C_{S}=C_{So}$.

### 3.2. $P_{v_{j}}^{o}\gamma_{j}$ Estimation and Correlation

The estimation of $P_{v_{j}}^{o}\gamma_{j}$ of each SVCOC *j* was carried out from data obtained in the surfactant presence in set B2 summarized in Table 1. The vapour–liquid equilibrium (VLE) of the component *j* can be described by modified Raoult’s law Equation (3), assuming the vapour phase is an ideal gas phase and the effect of the surfactant and VCOCs in the liquid phase is taken into account with the values of the product of the vapour pressure, $P_{v_{j}}$, and the activity coefficient, $\gamma_{j}$.

(3) $$P_{T}\cdot y_{j}=P_{v_{j}}^{o}\cdot \gamma_{j}\cdot x_{j}$$

where $P_{T}$ is the total pressure in the vial (bar) at the temperature T, *y_j_* is the molar fraction of chlorinated organic compound *j* in the vapour phase; $P_{v_{j}}^{o}$ is the saturation vapour pressure (bar) of compound *j*; *x_j_* is the molar fraction of compound *j* in the liquid phase; $\gamma_{j}$ is the activity coefficient of the *j* compound. 

In Equation (3), the total pressure in the vial (bar) is calculated assuming that at the conditions tested water and air are the main compounds in the gas phase in the vial, according to Equation (4).

(4) $$P_{T}\approx P_{air}+P_{w}=P_{o\;air}+P_{w\;\left(T\right)}^{o}\;$$

where $P_{T}$ is the sum of the air pressure ($P_{air},\;bar$) which can be considered the atmospheric pressure at 20 °C ($P_{o\;air},\;bar$) and water pressure ($P_{w},\;bar$) which is equals to water vapour pressure at T ($P_{W\left(T\right)}^{0},\;bar$), assuming that the molar fraction of water in the liquid phase is almost the unity. 

In the literature, there is scarce information regarding how the surfactants modify the ideality of the liquid phase. For that, experimental values of $P_{v_{j}}^{o}\gamma_{j}$ for each compound *j* at different temperatures, surfactant, and SVCOCs concentrations in the liquid phase were determined according to Equation (5), after measuring the gas phase composition of the vial by GC, as explained in SVCOCs analysis section.

(5) $$P_{v_{j}^{o}}\gamma_{j}\approx \frac{\frac{n_{j}}{n_{gas}}P_{T}}{x_{j}}\;$$

where $n_{j}$ is the moles of *j* compound in the vial gas phase, $n_{gas}$ is the sum of moles of all compounds (including organic, air, and water) in the vial gas phase, respectively, $P_{T}$ is the total pressure (bar) calculated with Equation (4), and $x_{j}$ is the molar fraction of the compound j in the liquid phase.

The experimental results of the $\ln\left(P_{v_{j}}^{o}\gamma_{j}\right)$ are shown in Figure S1. The effect of temperature, SVCOCs, and surfactant concentrations on the value of $\ln\left(P_{v_{j}}^{o}\gamma_{j}\right)$ were studied. The red points correspond to the values of $\ln\left(P_{v_{j}}^{o}\gamma_{j}\right)$ under different conditions. As can be seen, SVCOCs concentration did not affect $\ln\left(P_{v_{j}}^{o}\gamma_{j}\right)$ values at the same surfactant concentration and temperature. For this reason this variable was not taken into account in the estimation of $\ln\left(P_{v_{j}}^{o}\gamma_{j}\right)$. On the other hand, the higher the temperature, the higher the $\ln\left(P_{v_{j}}^{o}\gamma_{j}\right)$ values under the same conditions of surfactant concentration. In this way, the compounds have a major tendency to pass to the vapour phase. Lastly, regarding surfactant concentration, when keeping a constant temperature, the values of $\ln\left(P_{v_{j}}^{o}\gamma_{j}\right)$ decrease with the increase in C_S._ By raising the surfactant concentration, a higher concentration of micelles is generated [5], which results in the DNAPL being more protected. Therefore, the higher the surfactant concentration, the lower the volatilization of the compounds.

The interaction between surfactant concentration and temperature to $\ln\left(P_{v_{j}}^{o}\gamma_{j}\right)$ was modelled using the response surface methodology (RSM). In the RSM, the parameters in Equation (6) were fitted to the experimental $\ln\left(P_{v_{j}}^{o}\gamma_{j}\right)$ data in Figure S1.

(6) $$P_{v_{j}^{o}}\gamma_{j}=\exp\left(a+b\cdot C_{S}+c\cdot T+d\cdot C_{S}{}^{2}+e\cdot T^{2}+f\cdot C_{S}\cdot T\right)$$

where *T* is the temperature (°C), a-f are the parameters obtained from response surface methodology (Figure S1), and *C_S_* is the surfactant concentration ($g\cdot L^{-1}$) when VLE is reached (1 h). $C_{S}$ is the initial surfactant concentration since the surfactant does not lose capacity.

The results of the parameters *a-f* in Equation (6) and the statistical parameters obtained from the analysis of variance (Coefficient of variation (R^2^), Fischer’s test value (F-value), and probability (*p*-value)) obtained from the fitting are summarized in Table 2. As can be seen, the value of R^2^ is close to one for all the compounds present in SVCOCs, indicating the good compromise between the data obtained by experiments and those predicted by the model. Additionally, the F-values are large (>>1), and the p-values are small enough (<0.05) for the compounds, then, the model proposed for $P_{v_{j}}^{o}\gamma_{j}$ estimation can be considered valid and the values can be estimated accurately for a given surfactant concentration and temperature regardless of initial SVCOCs concentration.

### 3.3. Volatilization of SVCOCs from Emulsion

Volatilization of SVCOCs in the emulsion can be modelled considering those values that influence the volatility of the chlorinated organic compounds. These variables are temperature, airflow, and surfactant concentration in the aqueous phase. The molar balance of each chlorinated organic compound *j* in the emulsion in the batch experiment schematized in Figure 1 can be calculated using Equation (7).

(7) $$-\frac{dn_{j}}{dt}=-\frac{V_{L}C_{T}dx_{j}}{dt}$$

where $n_{j}$ is the moles of *j* in the emulsion; $V_{L}$ is the volume of the aqueous emulsion (L); $C_{T}$ is the total molar concentration of the emulsion (approximately corresponding to water: 55 $\mathrm{mol}\cdot L^{-1}$), and $x_{j}$ is the molar fraction of the compound *j* in the liquid phase.

The gas flow rate which leaves the bottle (Figure 1) is assumed to be in equilibrium with the emulsion by applying Raoult’s law. The molar fraction of *j* compound in the gas phase is calculated with Equation (8).

(8) $$y_{j}=\frac{P_{v}^{o}\gamma_{j}\cdot x_{j}}{P_{T}}$$

The molar flow rate of the *j* compound disappearing from the emulsion is the same as the molar flow of this *j* compound that leaves the bottle in the gas phase (both phases in equilibrium), as described in Equation (9).

(9) $$-\frac{V_{L}\cdot C_{T}\cdot dx_{j}}{dt}=\frac{F_{gas}\cdot P_{v}^{o}\gamma_{j}\cdot x_{j}}{P_{T}}$$

where $F_{gas}$ is the gas molar flow rate ($\mathrm{mol}\cdot h^{-1}$) fed to the system.

The molar fraction of *j* in the emulsion with time can be predicted by integrating Equation (9) as shown in Equation (10), where K is a constant defined in Equation (11).

(10) $$\frac{x_{j}}{x_{jo}}=\exp\;(-K_{j}\cdot t)$$

(11) $$K_{j}=\frac{F_{gas}\cdot P_{v_{j}}^{o}\gamma_{j}}{V_{L}\cdot C_{T}\cdot }$$

The value of $P_{v}^{o}\gamma_{j}$ at each time is obtained by Equation (6), considering that surfactant concentration is the initial one since it keeps constant with the time.

The ratio $x_{j}/x_{jo}$ also corresponds to the concentration ratio of *j* compound in the emulsion (Equation (12)).

(12) $$\frac{x_{j}}{x_{j0}}=\frac{C_{j}}{C_{j0}}$$

**Figure 3:**
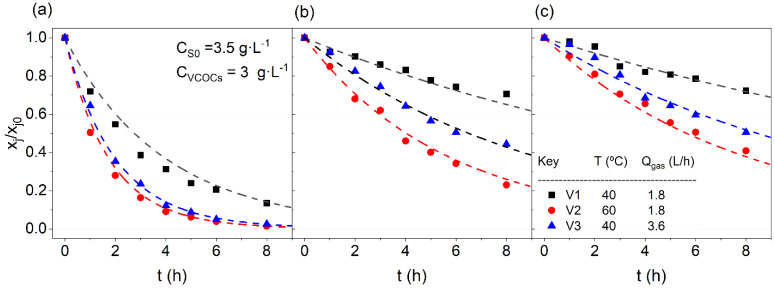
Volatilization of SVCOCs in the emulsion for (**a**) CB; (**b**) 1,4-DCB; (**c**) 1,2-DCB. Conditions C_SVCOCs_ = 23.5 $\mathrm{mmol}\cdot L^{-1}$; C_S0_ = 3.5 $g\cdot L^{-1}$; V_aq_ = 0.3 L. SVCOCs distribution (as molar percentage) was 53% of CB, 29% of 1,2-DCB, and 18% of 1,4-DCB. Symbols depict experimental results and line values predicted using Equation (10).

The consistency of $P_{v_{j}^{o}}\gamma_{j}$ obtained in the surfactant presence was validated by comparing the experimental and predicted values of each compound in the emulsion obtained in runs in Table 1 (set B3). Experimental values with time of each time in emulsion (as symbols) and those predicted with Equation (10) (as lines) are shown in Figure 3.

In Figure 3, either experimental values (as symbols) or simulated ones (as lines) have been plotted. As can be seen, the values of $x_{j}/x_{j0}$ for each compound j (j = CB, 1,4-DCB, 1,2-DCB) are in good agreement with the experimental results, so the model is validated. 

The effects of temperature and flow of air were studied. Experiments V1 and V2 were carried out at different temperatures. The higher the temperature (V2 experiment), the lower the fraction of SVCOC that remained in the aqueous emulsion, as was shown in Figure 3. Under the most favourable temperature conditions (60 °C), the CB removal was completed, whilst the values for 1,4-DCB and 1,2-DCB were 0.23 and 0.41, respectively. The temperature increase from 40 to 60 °C yielded an improvement of 89, 67, 44% value of the remaining ratio of SVCOCs after 8 h of volatilization treatment.

On the other hand, experiment V3 was carried out using a two-fold air flow rate, compared to the one used in V1. This variable presented a lower impact than the temperature increase in the remaining ratio of SVCOCs after 8 h. The improvement performed using a higher air flow were 81, 37, and 30% for CB, 1,4-DCB, and 1,2-DCB, respectively. As concerns each SVCOC, chlorobenzene is the compound that is more easily volatilized because it has the highest value of $P_{v}^{o}\gamma$ (Figure S1), reaching almost complete elimination of the emulsion for experiments V2 and V3 in 8 h of treatment. For 1,2-DCB and 1,4-DCB, the results obtained are very similar between them, where the volatilization treatment was slightly more efficient for 1,4-DCB because of its slightly higher values of $P_{v}^{o}\gamma$ (Figure S1). After 8 h of aeration, the values reached were 0.2 for 1.4-DCB and 0.3 for 1,3-DCB for the V3 experiment. Therefore, to obtain an increase in the volatilization of SVCOCs, ot is necessary to use higher temperature and air flow, controlling the surfactant capacity loss and costs. 

**Figure 4:**
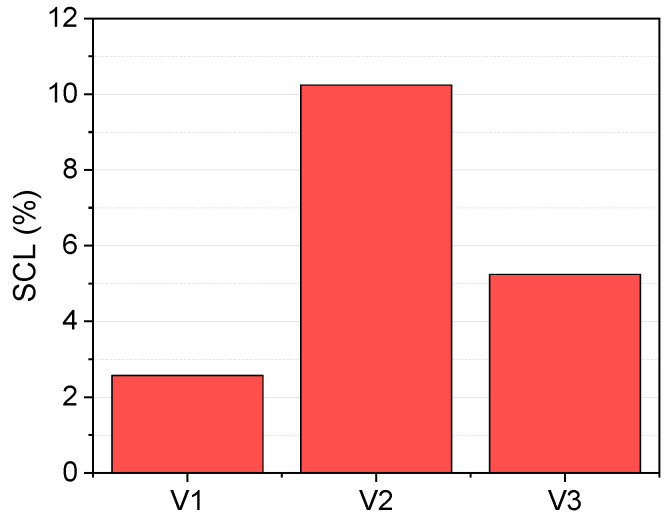
SCL values for each volatilization experiment.

The surfactant capacity loss (SCL) during volatilization has also been measured and estimated for runs in Table 1. These aim to analyze if the surfactant can be reused in SEAR remediation treatments. The results obtained are summarized in Figure 4:

As observed in Figure 4, the SCL caused in the SVCOCs air stripping is lower than 12% for all the experiments, indicating that the surfactant can be reused. This surfactant would be employed in a new cycle of SEAR treatment (in situ), reducing the operational costs. The use of E-Mulse^®^ in SEAR treatment has been successfully applied in a real soil polluted with DNAPL. This DNAPL is formed by a complex mixture of chlorinated compounds among which are chlorobenze and dichlorobenzene isomers [8].

The emulsion extracted from the new SEAR cycle will be treated by SVCOCs volatilization, which will have better results since the $P_{v}\gamma$ values increase with a decrease in surfactant concentration. However, it is important to point out that with each new cycle of volatilization, the surfactant capacity will decrease around 10%, so the dissolved SVCOCs in the next SEAR treatment will also decrease, taking into account that the MSR is 4.33 mmol SVCOCsg surfactant. This process can be repeated until the quantity of SVCOCs dissolved does not support the reuse of the surfactant. 

It is important to point out that the volatilization process results forms the need to treat the resulting effluent after SEAR treatment, as it contains chlorinated compounds that require elimination. 

## 4. Conclusions

In this work, the mixture of semi-volatile chlorinated organic compounds has been successfully volatilized from a non-ionic surfactants emulsion, reducing remarkably the concentration of SVCOCs in the emulsion, but keeping the surfactant capacity for recycling the emulsion in further SEAR treatments.

Regarding surfactant stability, it has been observed that surfactant capacity keeps constant at temperatures up to 60 °C during 48 h, with Surfactant Capacity Loss lower than 10%.

From the experimental results, the thermodynamic behavior of the SVCOCs in the emulsion was remarkably affected by surfactant concentration and temperature. $P_{vj}^{o}\gamma_{j}$ estimated values were correlated with surfactant concentration and temperature using surface response methodology. SVCOCs concentration does not affect the $P_{v}^{o}\gamma$ values. It has been concluded that $P_{v}^{o}\gamma$ is increased with the temperature due to the significant tendency of the organic compounds to pass to the vapour phase and reduce with the surfactant concentration.

The model proposed to simulate the evolution of SVCOCs in the emulsion during the air stripping process was successfully to predict the experimental values. Therefore, the estimated $P_{vj}^{o}\gamma_{j}$ values were validated. The SCL, after eliminating more than 80% of COCs in the emulsion, was lower than 10%, and the resulting emulsion could be used in further soil flushing in a circular economy scenario.

## Figures and Tables

**Table 1:** Experimental conditions for the experimental set. SVCOCs distribution (as molar percentage) was 53% of CB, 29% of 1,2-DCB, and 18% of 1,4-DCB.

Set B1					
**Exp**	**T (°C)**	$C_{S0}\;(g\cdot L^{-1})$		***C_SVCOCs_* (** $mmol\cdot L^{-1}$ **)**	
**E1**	20	10		0	
E2	40	10		0	
E3	60	10		0	
E4	20	10		94.11	
E5	40	10		94.11	
E6	60	10		94.11	
Set B2					
**Exp**	**T (°C)**	$C_{S0}\;(g\cdot L^{-1})$		***C_VCOCs_* (** $mmol\cdot L^{-1}$ **)**	
P1	30, 40, 60	1.5		3.1, 6.3	
P2	30, 40, 60	3.5		7.5, 19.6	
P3	30, 40, 60	7.0		7.8, 23.5, 39.2	
P4	30, 40, 60	15.0		15.6, 31.3, 62.8	
		Set B3			
**Exp**	**T (°C)**	$C_{S0\;}(g\cdot L^{-1})$	$C_{SVCOCs\;}(mmol\cdot L^{-1})$	$Q_{gas}\;(L\cdot h^{-1})$	***V emulsion* (L)**
V1	40	3.5	23.5	1.8	0.3
V2	60	3.5	23.5	1.8	0.3
V3	40	3.5	23.5	3.6	0.3

**Table 2:** Parameters obtained from the fitting of $P_{v_{j}}^{o}\gamma_{j}$ to Equation (6). The statistical parameters were obtained from variance analysis. Coefficient of variation (R^2^), Fischer’s test value (F-value), and probability (*p*-value) are also shown.

	*a*	*b*	*c*	*d*	*e*	*f*	$R^{2}$	F-Value	*p*-Value
CB	2.86	−0.27	0.06	8.73 × 10^−3^	−1.32 × 10^−4^	−6.74 × 10^−5^	0.99	414	1.86 × 10^−22^
1,4-DCB	0.29	−0.30	0.09	1.21 × 10^−2^	−2.85 × 10^−4^	−8.10 × 10^−4^	0.99	473	3.73 × 10^−23^
1,2-DCB	0.31	−0.30	0.08	1.19 × 10^−2^	−2.34 × 10^−4^	−8.13 × 10^−4^	0.99	666	6.52 × 10^−25^

## Data Availability

Not applicable.

## Supplementary Materials

The following supporting information can be downloaded at: https://www.mdpi.com/article/10.3390/ijerph19127547/s1, Table S1. HS-GC conditions. Values of $P_{v_{j}}^{o}\gamma_{j}$ for the experiments summarized in Table S1 (red points) and the response surfaces for the different compounds.
