# Supplementary material for: Non-Ionic Surfactant Recovery in Surfactant Enhancement Aquifer Remediation Effluent with Chlorobenzenes by Semivolatile Chlorinated Organic Compounds Volatilization
Source: Int J Environ Res Public Health. 2022 Jun 20;19(12):7547. doi: 10.3390/ijerph19127547 (PMC9223721; doi:10.3390/ijerph19127547)
Supplement: Supplementary file 1 [file ijerph-19-07547-s001.zip › ijerph-1756418-supplementary.pdf]

International Journal of
Environmental Research and Public Health

MDPI

Non-Ionic Surfactant Recovery in Surfactant Enhancement Aquifer Remediation Effluent with Chlorobenzenes by Semivolatile Chlorinated Organic Compounds Volatilization

Patricia Sáez [1], Aurora Santos [1], Raúl García-Cervilla [1], Arturo Romero [1] and David Lorenzo [1,*]

[1] Chemical Engineering and Materials Department, Complutense University of Madrid., Madrid 28040, Spain.
* Correspondence: dlorenzo@quim.ucm.es

SUPPLEMENTARY MATERIAL

Table S1. HS-GC conditions.

Parameters	Conditions
Headspace	
Incubation Temperature (ºC)	30, 40, 60
Incubation time (min)	60
Syringe Temperature (ºC)	90
Agitator Speed (r.p.m.)	500
Gas Cromatograph	
Carrier gas	Helium
Carrier gas pressure (kPa)	22.7
Injector Temperature (ºC)	180
Injection Volume (mL)	2.5
Split Ratio	10:1
Split Flow (mL·min^{-1})	50
Temperature Program	Initial Temperature 80 ºC<br>Ramping to 80 ºC at 180ºC hold for 9 min
Detectors FID	Heater: 300ºC<br>Air Flow: 300 mL/min<br>H_2 Fuel Flow: 40 mL/min<br>Makeup Flow N_2: 40 mL/min
Detectors ECD	Heater: 210ºC<br>Makeup Flow N_2: 40 mL/min

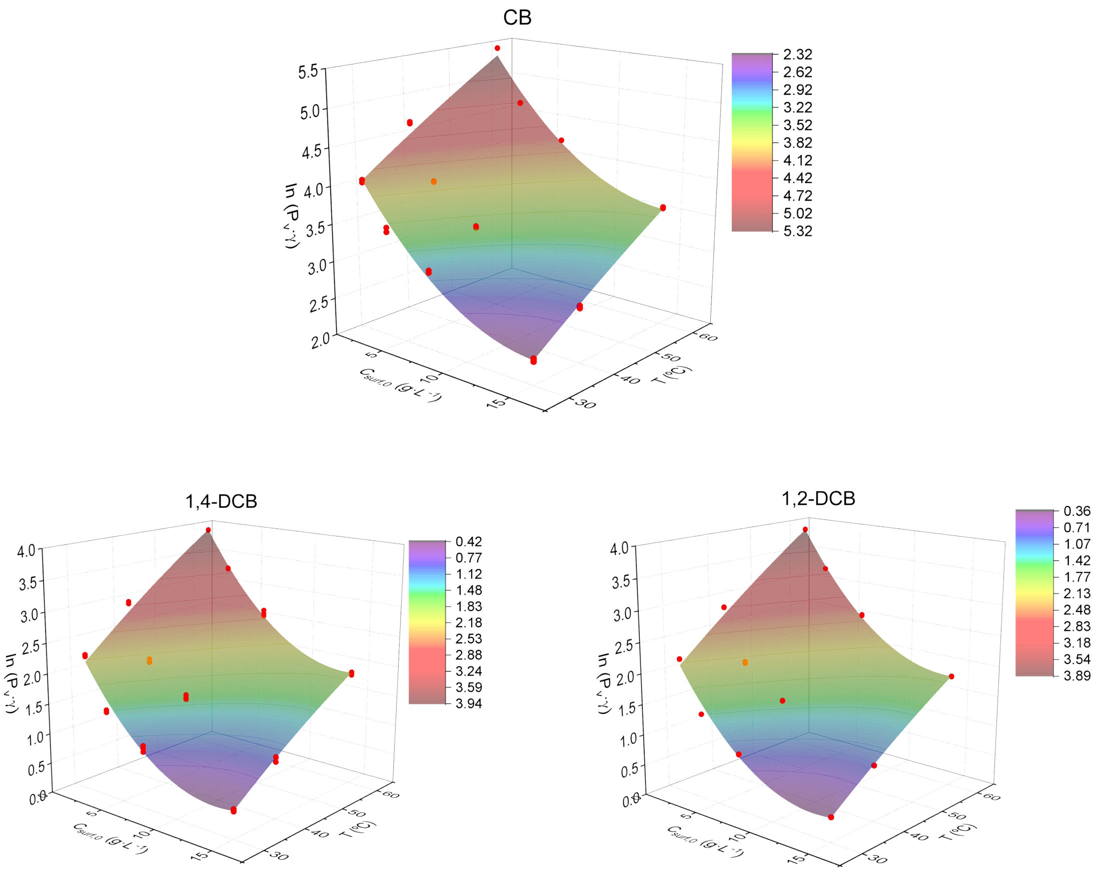

Figure S1. Values of $P_{v_j}^{o}\gamma_j$ for the experiments summarized in Table S1 (red points) and the response surfaces for the different compounds
